# Imaging of Airway Obstruction in Children

**DOI:** 10.3389/fped.2020.579032

**Published:** 2020-11-11

**Authors:** Derek J. Roebuck, Conor Murray, Clare A. McLaren

**Affiliations:** ^1^Department of Medical Imaging, Perth Children's Hospital, Nedlands, WA, Australia; ^2^Division of Pediatrics, Medical School, University of Western Australia, Crawley, WA, Australia; ^3^School of Molecular and Life Sciences, Curtin University, Bentley, WA, Australia

**Keywords:** imaging, radiology, airway obstruction, trachea - abnormalities, bronchus, computed tomography, magnetic resonance imaging, tracheobronchomalacia

## Abstract

Various imaging techniques may be used to diagnose airway obstruction in children. Digital radiography, computed tomography and magnetic resonance imaging are the most important modalities, but the choice of technique will depend on the level and nature of suspected obstruction, as well as patient-specific factors such as age and ability to cooperate. This review examines the forms of airway obstruction that are commonly encountered in childhood.

## Introduction

Airway obstruction may occur at any point from the nostrils to the bronchioles, and different imaging modalities may be appropriate at each level. The structure of the larger airways is relatively easy to image, although special techniques may be required to show dynamic obstruction. Small airway obstruction is often only shown by indirect imaging evidence, especially the presence of air trapping.

This article will review the non-invasive imaging techniques used in current practice and the imaging findings of some of the disease processes that may cause airway obstruction in childhood.

## Imaging Techniques

**Figure 1:**
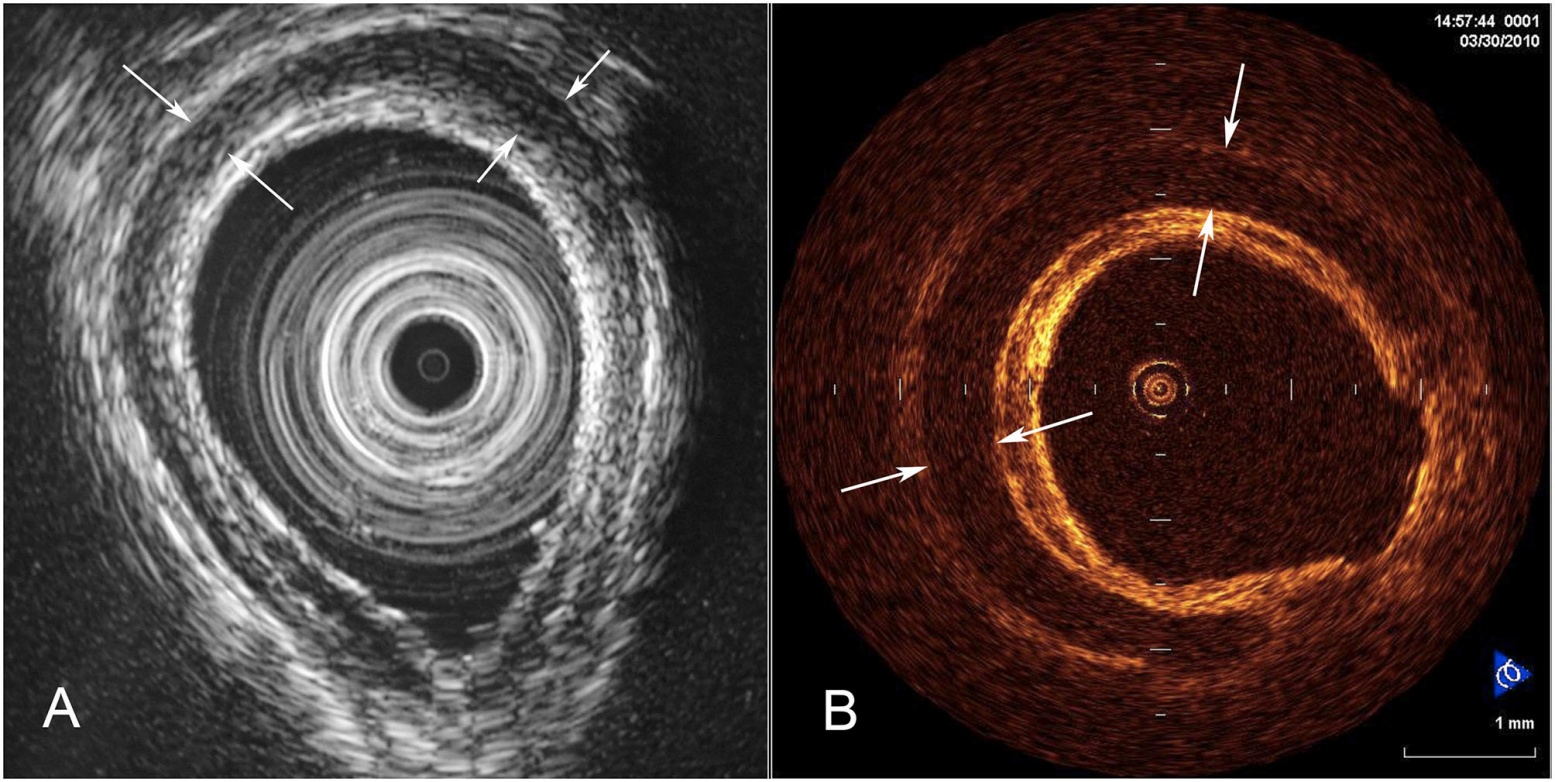
Endoluminal imaging techniques. **(A)** Endobronchial ultrasound image showing a cross-section of tracheal wall. The tracheal cartilage is shown as a dark (hypoechoic) band. **(B)** Corresponding optical coherence tomography image (from a different patient) showing the structure of the tracheal cartilage.

Various techniques may be used to image airway pathology in children (Table 1). The most versatile of these is computed tomography (CT), but magnetic resonance imaging (MRI), ultrasound, digital radiography and fluoroscopic examinations may also be useful. The use of certain nuclear medicine techniques has been investigated, but these have not been widely adopted (1). An exception is the use of positron emission tomography to characterize and stage tumors. Endobronchial ultrasound is rarely used in children (2), even though high-quality images can be obtained (Figure 1A). Optical coherence tomography is a promising technique for high resolution imaging (Figure 1B), but also requires instrumentation of the airway. Bronchography is invasive and arguably non-physiological, and is now used mainly as part of interventional procedures rather than purely for diagnosis (3).

**Table 1:** Major techniques for imaging of airway obstruction in children.

**Technique**	**Relevant applications**	**Advantages**	**Disadvantages**
Ultrasound	Neck imaging (e.g., vocal cords)	Cheap and mobile	Not useful for direct imaging of intrathoracic airways
	Echocardiography (e.g., vascular compression)	Real-time imaging	
Radiographs	Upper airway imaging (e.g., adenoidal hypertrophy)	Cheap and mobile	Poor soft tissue contrast resolution
	Thoracic imaging		Poor depiction of smaller airways
			Not useful for tracheobronchomalacia
Computed tomography (CT)	Upper airway imaging (e.g., choanal atresia)	Good soft tissue contrast resolution	Radiation dose concerns (especially dynamic imaging)
	Neck imaging (e.g., retropharyngeal abscess)	Large and medium airways directly shown	
	Large intrathoracic airway imaging	Detects air trapping (small airways disease)	
	Obstruction at the small airway level	Possibility of dynamic imaging Sedation rarely required with modern equipment	
Magnetic resonance imaging	Upper airway imaging	As for CT (currently under evaluation)	Possible need for sedation or general anesthesia
	Large intrathoracic airway imaging		Poor temporal resolution for dynamic imaging

## Levels of Obstruction

### Supraglottic Airway Obstruction

Nasal and/or pharyngeal obstruction may be isolated, or may co-exist with and exacerbate lower airway obstruction. Nasal obstruction (for example by piriform aperture stenosis or choanal atresia) is well-demonstrated at CT, which may be useful to determine the level and nature of the obstruction, and to look for other features of syndromes (such as CHARGE association) which may cause choanal atresia (4).

Although adenoidal and tonsillar hypertrophy can be shown on a lateral radiograph (5), the exact contribution of this test to clinical decision making is unclear. Imaging is not a routine part of the assessment of children with sleep-disordered breathing (6, 7).

Although numerous other congenital conditions can contribute to upper airway obstruction, the main role of imaging is typically in pre- and post-operative evaluation (8, 9), and is beyond the scope of this article.

Radiographs can demonstrate some foreign bodies at the level of the pharynx, for example certain fish or other bones, although interpretation of the images can be difficult (10, 11). Pharyngeal and esophageal foreign bodies rarely cause airway symptoms unless complications such as abscess or airway fistula have developed. Although retropharyngeal abscesses can be detected with ultrasound, CT is usually preferred because it better demonstrates the extent of infection and allows for surgical planning (12).

### Laryngeal and Subglottic Obstruction

Airway obstruction at the level of the larynx and subglottis is conventionally evaluated by endoscopy. Radiographs of the airway were historically used to diagnose acute epiglottitis and croup (5), but the potential clinical benefit of this is very small. Additionally, imaging may lead to a delay in appropriate treatment, and is therefore not a part of current guidelines for routine management of children with suspected croup. Compliance with guidelines is, however, variable. One study found that emergency departments staffed by non-pediatric specialists obtained radiographs in children with croup more often than those staffed by pediatricians (13).

Radiographs are, however, valuable in the detection of ingested metallic items such as button batteries, which may cause airway obstruction and masquerade as croup (14, 15).

**Figure 2:**
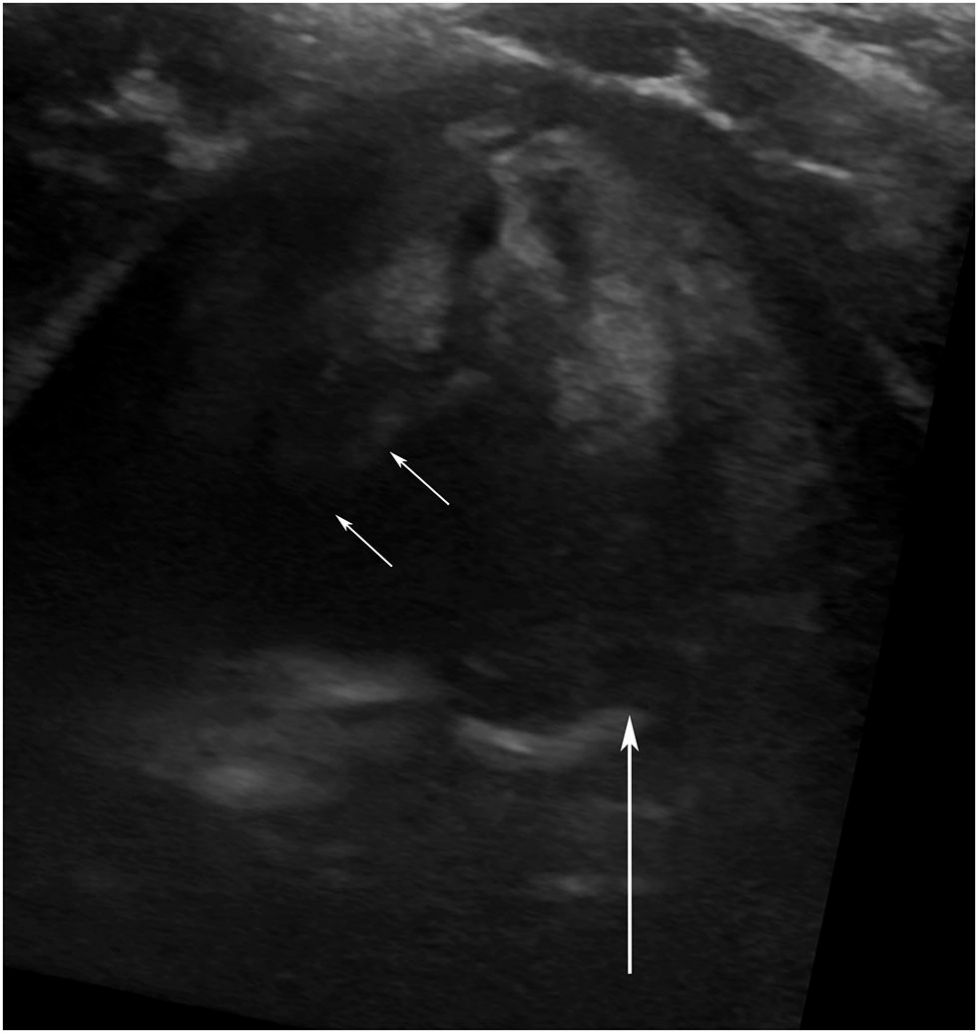
Vocal cord ultrasound. Transverse image of the larynx in a 3-year-old male 2 days after division of a vascular ring. The right vocal fold (small arrows) is abducted. The edge of the left vocal fold is hard to visualize (because it runs parallel to the ultrasound beam), but its adducted position is confirmed by the medial position of the left arytenoid cartilage (large arrow).

Conventional (transcutaneous) ultrasound can also be used to image the larynx and upper trachea. Currently, the principal application is non-invasive evaluation of vocal cord movement (Figure 2) (16, 17). Very high resolution images can be obtained of the thyroid, cricoid, and the first few tracheal cartilages (18). Ultrasound is also a promising technique for the non-invasive diagnosis of laryngomalacia (19) and vallecular cysts (20). Although laryngeal function can also be evaluated with CT (21), its use has not become popular in children, perhaps because of concerns about radiation dose to the thyroid gland.

### Tracheobronchial Obstruction

**Figure 3:**
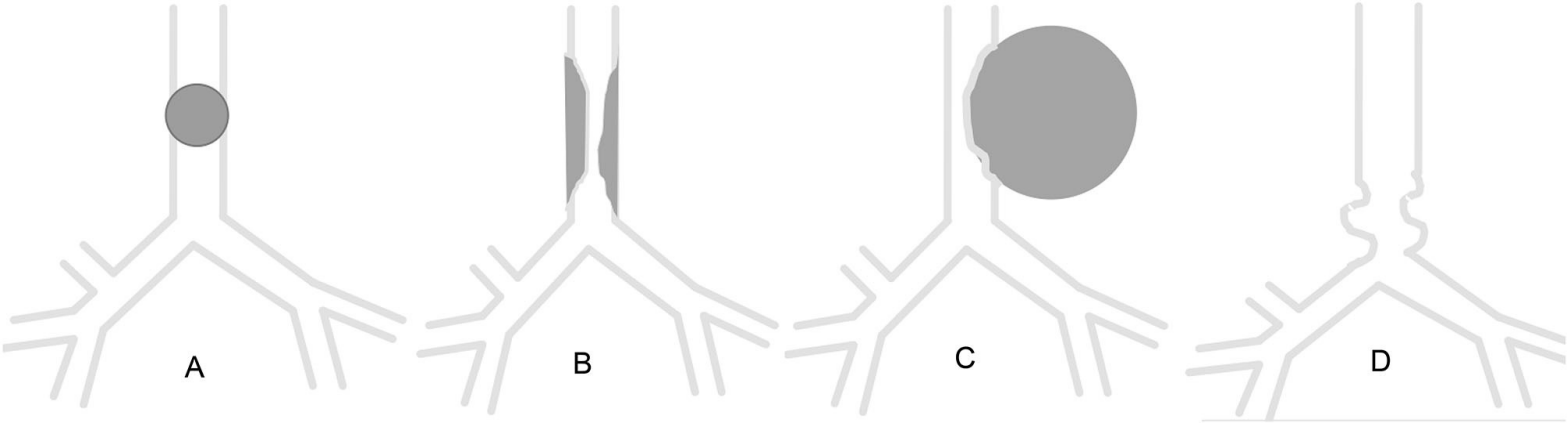
Schematic of patterns of major airway obstruction. **(A)** Intraluminal obstruction. **(B)** Congenital or acquired stenosis. **(C)** Extrinsic compression. **(D)** Tracheobronchomalacia.

A simplified approach to obstruction of the major thoracic airways identifies four principal mechanisms, although occasionally more than one is present (Figure 3). The clinical challenge is to use the appropriate combination of structural, functional, and indirect imaging to assess the suspected cause in each patient. CT is usually the best adjunct to bronchoscopy, especially when more than one mechanism of obstruction is suspected (22).

### Congenital Tracheal Stenosis

**Figure 4:**
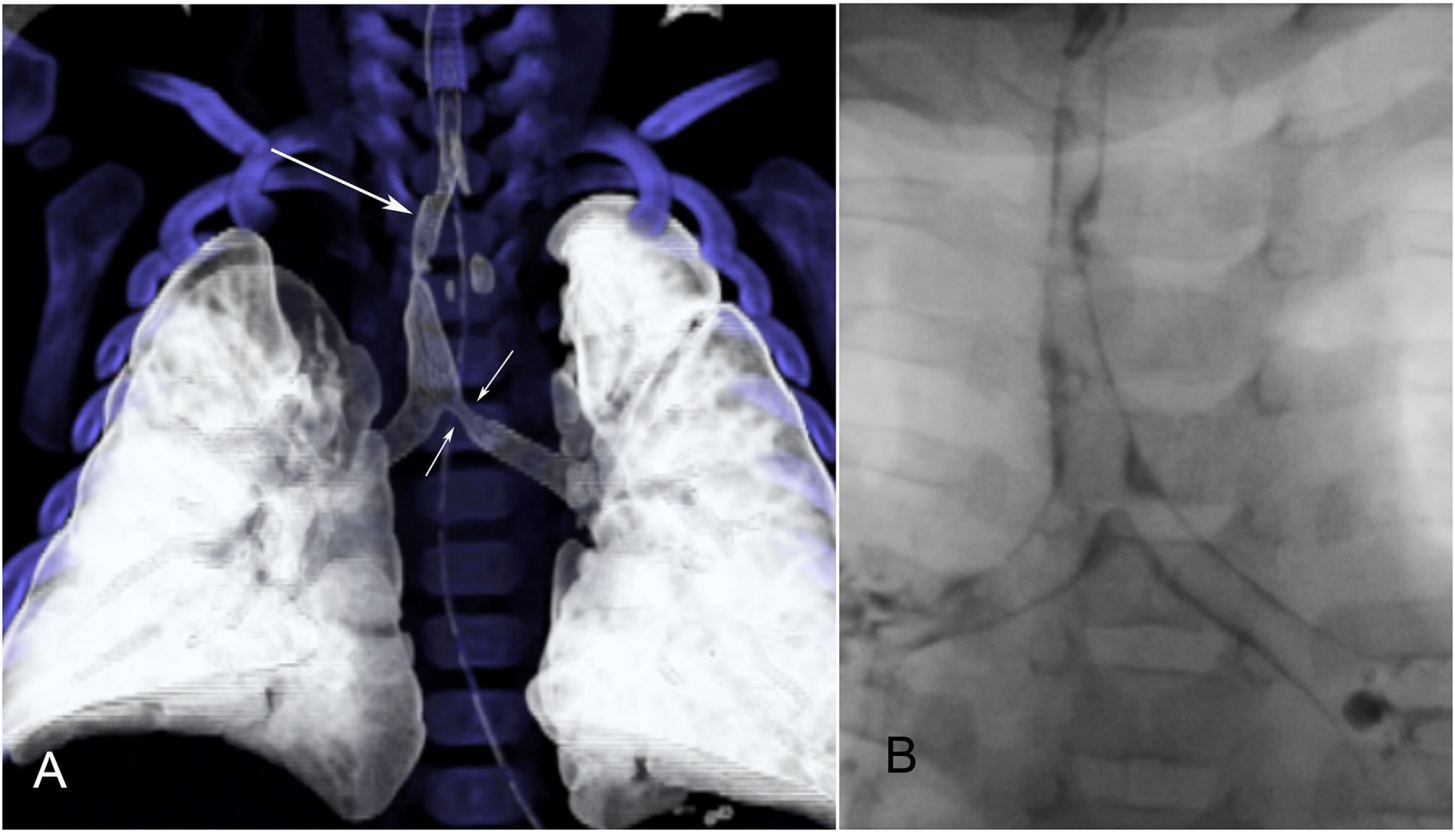
Congenital tracheal stenosis with bronchomalacia. **(A)** Reformatted CT image shows the congenital stenosis (long arrow) quite well. A second area of narrowing is present at the proximal end of the left bronchus (short arrows) on this single-phase study. **(B)** Dynamic bronchography shows that this is due to bronchomalacia and not a second stenosis.

CT and bronchoscopy are complimentary in the evaluation of children with suspected congenital tracheal stenosis. CT is particularly helpful for assessment of associated cardiovascular anomalies, such as aberrant left pulmonary artery, which are common. Care must be taken not to underestimate the extent of disease or misdiagnose associated bronchomalacia as fixed stenosis (Figure 4) (23). This is also a good example of the value of combining bronchoscopy with bronchography, which has excellent spatial and temporal resolution, and which demonstrates the airway distal to the stenosis, where it may not be safe to advance the bronchoscope (24). Modern CT scanners can overcome most of these problems using dynamic imaging techniques. CT allows accurate measurement of the length and diameter of involved airway segments, which is difficult with bronchoscopy, and may be crucial for planning management.

### Bronchial Atresia

**Figure 5:**
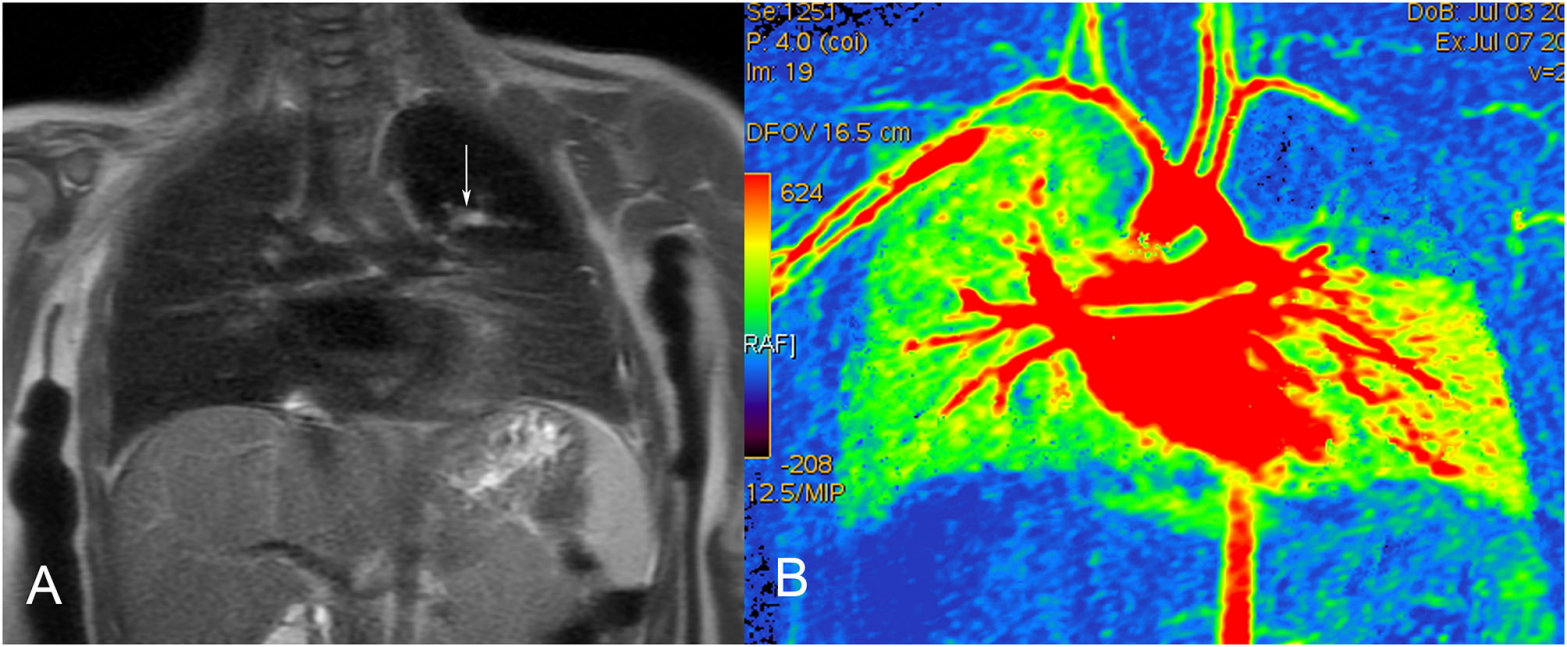
Bronchial atresia in a 12-month-old female. **(A)** MRI (T1-weighted coronal image) shows the mucocele as a branching hyperintense structure, extending into the abnormal left upper lobe. **(B)** Color-coded MRI perfusion image shows decreased flow to the affected lobe.

This uncommon congenital lesion occurs when there is interruption of the lumen of a lobar or segmental bronchus, usually associated with the development of a mucocele, as well as decreased attenuation (and often hyperexpansion) of the affected part of the lung (25). CT and MRI show a branching tubular structure, usually arising at or close to the hilum (Figure 5).

### Acquired Airway Stenosis

Most iatrogenic stenosis is diagnosed at bronchoscopy, but other causes of acquired stenosis (such as granulomatous disease) may be demonstrated first by imaging. The presence of other findings, for example hilar lymphadenopathy or cavitating lung lesions, may be a clue to the diagnosis.

### Inhaled Foreign Bodies

**Figure 6:**
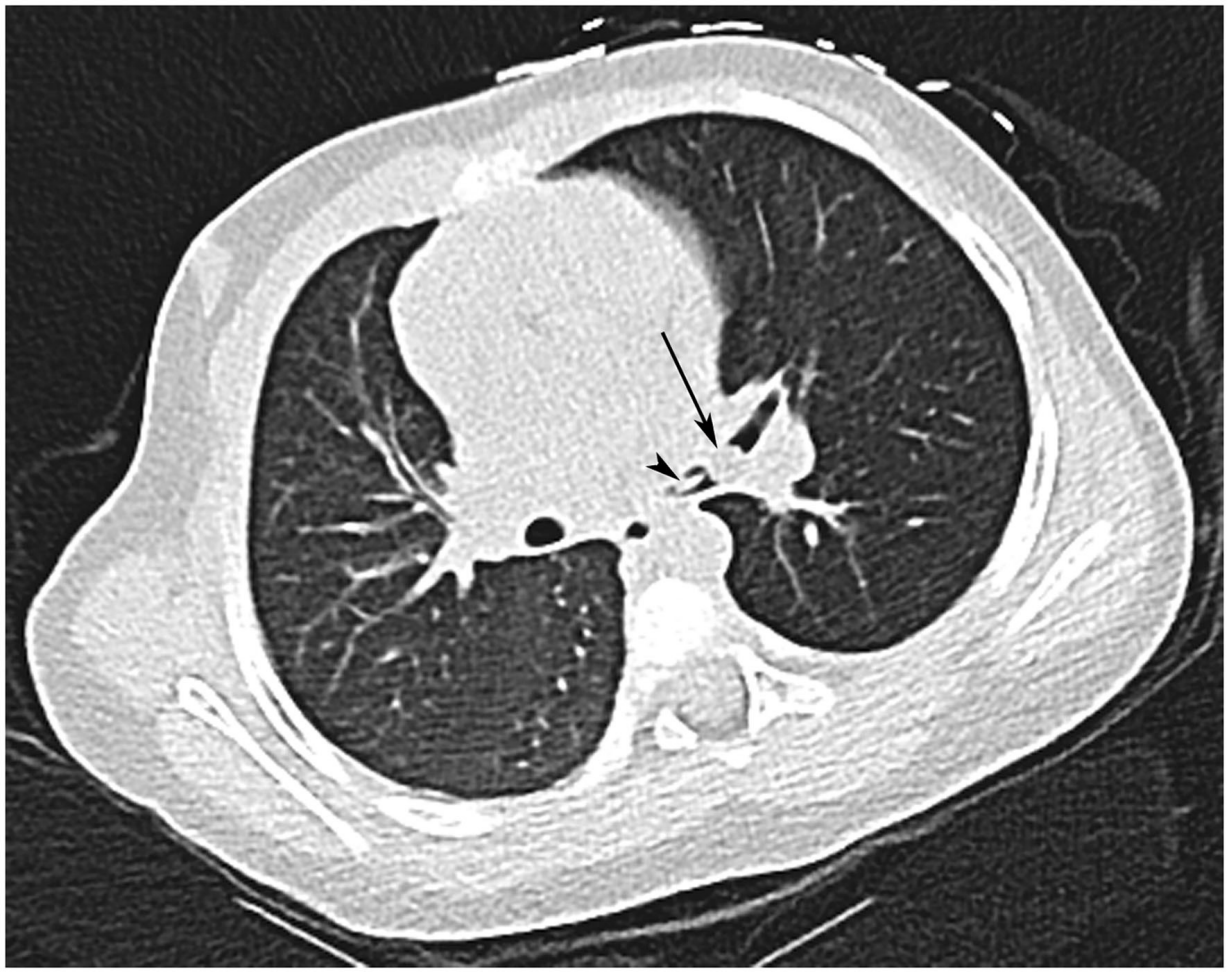
Inhaled foreign body in a 20-month-old female who choked on a piece of fruit. CT shows a fragment of fruit (arrow) and its stalk (arrowhead) at the bifurcation of the left main bronchus. Note also the decreased attenuation of the left lung.

Chest radiographs are the simplest method of demonstrating air trapping or lung collapse in children who are suspected of having aspirated a foreign body, although their sensitivity is quite poor (26). CT is more accurate, and may have a role in reducing the number of negative bronchoscopies for suspected foreign body (26, 27). When performed on modern equipment the radiation dose can be reduced to extremely low levels for this indication. CT may also facilitate bronchoscopy by directing the operator to the correct bronchus (Figure 6) (27).

### Endoluminal Tumors

**Figure 7:**
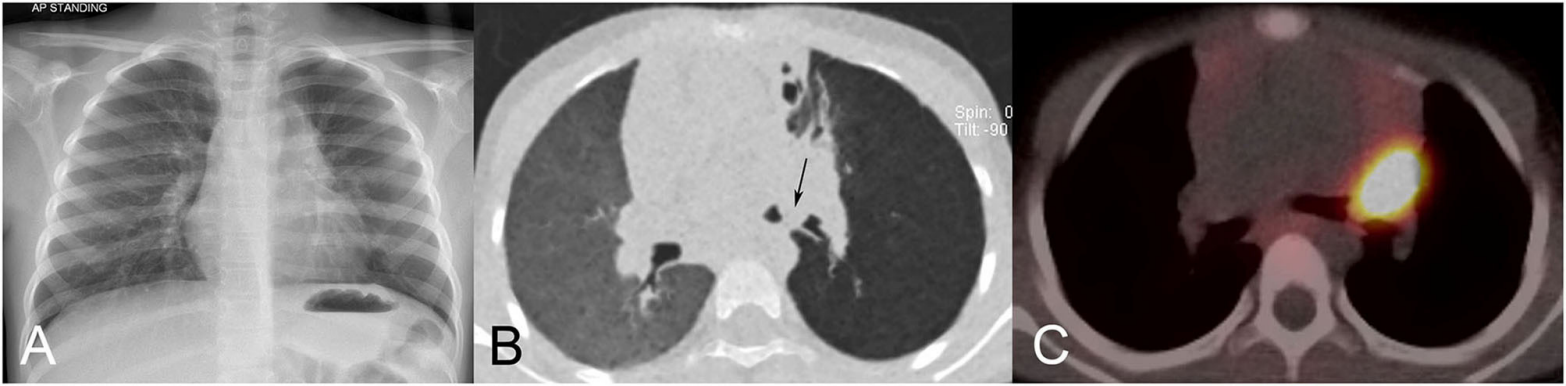
Carcinoid tumor in a 6-year-old-girl. **(A)** Chest radiograph shows a left hilar mass and hyperlucency of the left upper lobe. **(B)** Minimum intensity projection (MinIP) CT image shows a soft tissue mass occluding the left upper lobe bronchus, and protruding into the left bronchial bifurcation (arrow). Note the striking hypoattenuation (hyperlucency) of the left lung. **(C)** ^68^Ga-octreotate positron-emission tomography CT images shows strikingly high uptake in the lesion without evidence of metastatic disease.

The most commonly seen lesions in school age children are carcinoid tumor (Figure 7), inflammatory myofibroblastic tumor and mucoepidermoid carcinoma (28). In general, these are best evaluated with CT. Depending on the tumor type, positron emission tomography with ^18^F-fluorodeoxyglucose or ^68^Ga-octreotate may be appropriate to determine the extent of the disease (Figure 7).

### Vascular and Cardiac Compression

**Figure 8:**
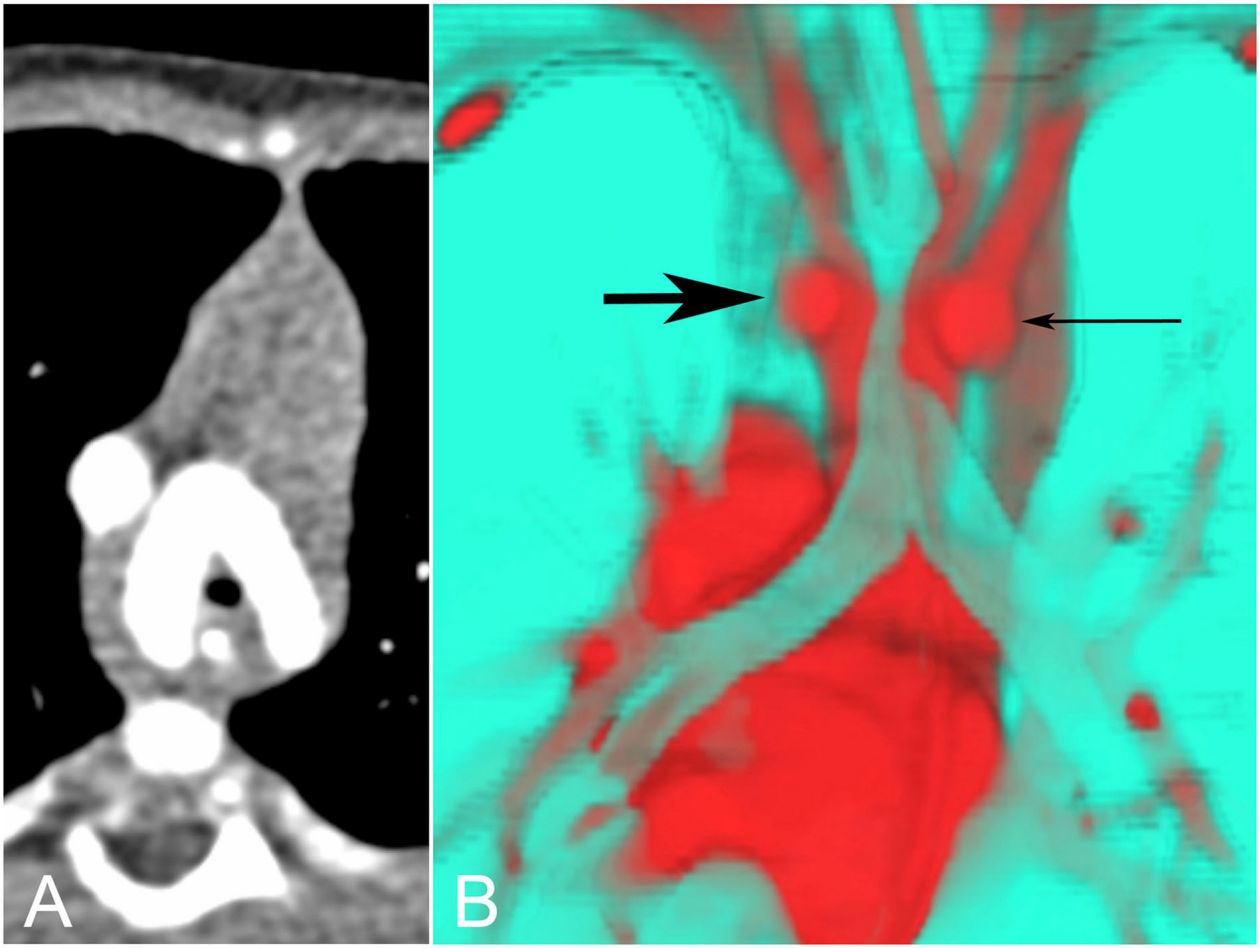
Tracheal compression by double aortic arch. **(A)** The tracheal lumen is narrowed as it passes between the aortic arches. **(B)** The same data can be presented in many other ways. Here a color-coded posterior view shows the trachea in turquoise, compressed by the left (thick arrow) and right (thin arrow) aortic arches in red.

Airway obstruction due to vascular compression occurs in about 1–2% of children with congenital heart disease, but is particularly common in certain types, and especially when there are anomalies of the great vessels (29). Compression may occur when the configuration of the great vessels is abnormal, such as double aortic arch (Figure 8), or when normally-positioned vessels are enlarged, as in absent pulmonary valve syndrome (29). Acquired conditions such as dilated cardiomyopathy may also cause airway obstruction (29).

In the past contrast studies of the esophagus were a mainstay of the evaluation of airway obstruction, but these are no longer used because CT provides much more information at a similar or even lower radiation dose.

### Other Extrinsic Compression

**Figure 9:**
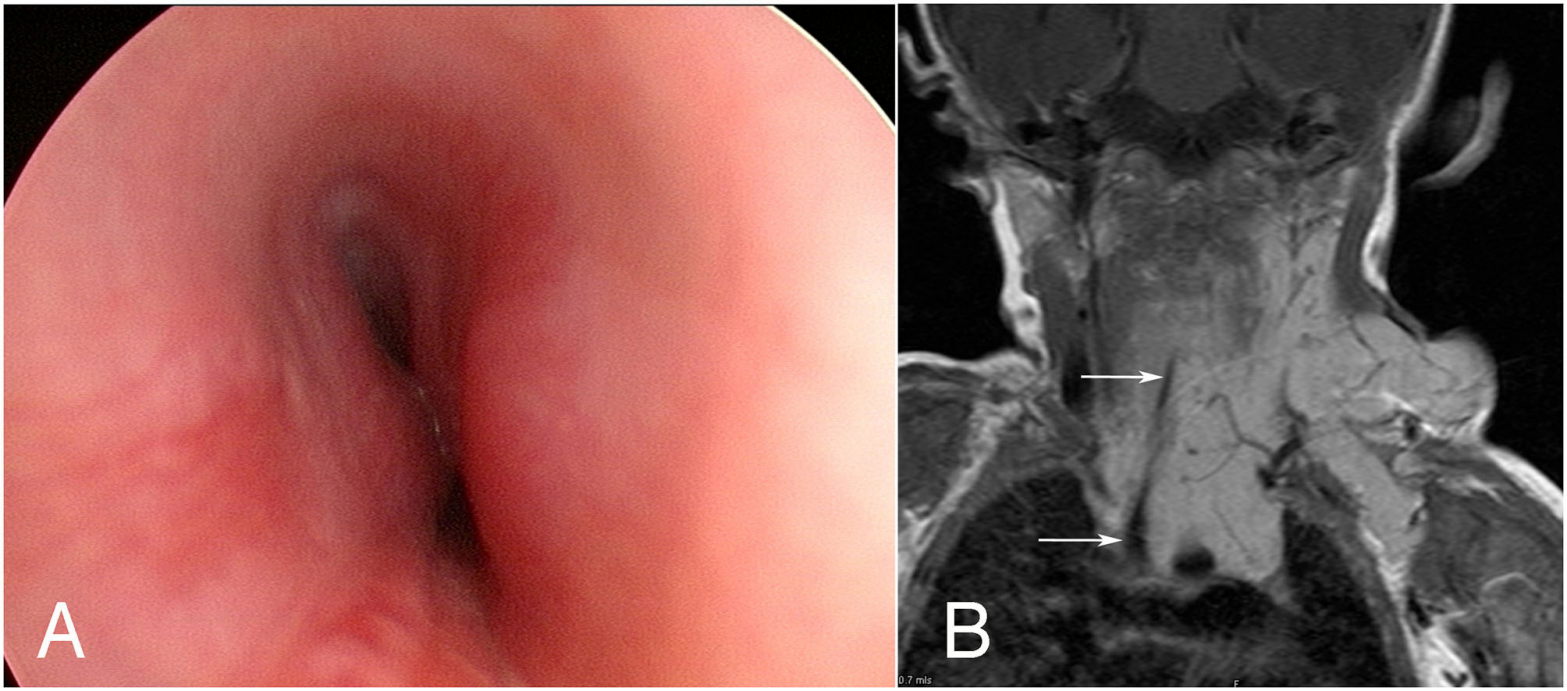
Tracheal compression by infantile hemangioma. **(A)** Bronchoscopy shows side-to-side compression of the upper trachea. **(B)** MRI (contrast-enhanced T1-weighted coronal image) shows the extent of the lesion in the neck, mediastinum and left axilla, as well as severe tracheal compression (arrows).

The airway may also be compressed by benign or malignant tumors or by vascular malformations (usually lymphatic). The most common benign tumor is an infantile hemangioma, which often causes compression in the subglottic region. These lesions are often difficult to evaluate fully with ultrasound, and MRI is preferred to show their full extent and to confirm the diagnosis. Bronchoscopy and cross-sectional imaging are complementary techniques in this respect (Figure 9).

The severe compression sometimes caused by other tumors such as lymphoma and neuroblastoma at first diagnosis usually responds quickly to chemotherapy, but recurrent tumor may be resistant to treatment and ultimately fatal. CT or MRI may be used to image these tumors. Multidisciplinary preoperative assessment is essential, because decisions about which tissue to biopsy (and anesthetic technique) may be quite complicated (30).

### Tracheobronchomalacia

**Figure 10:**
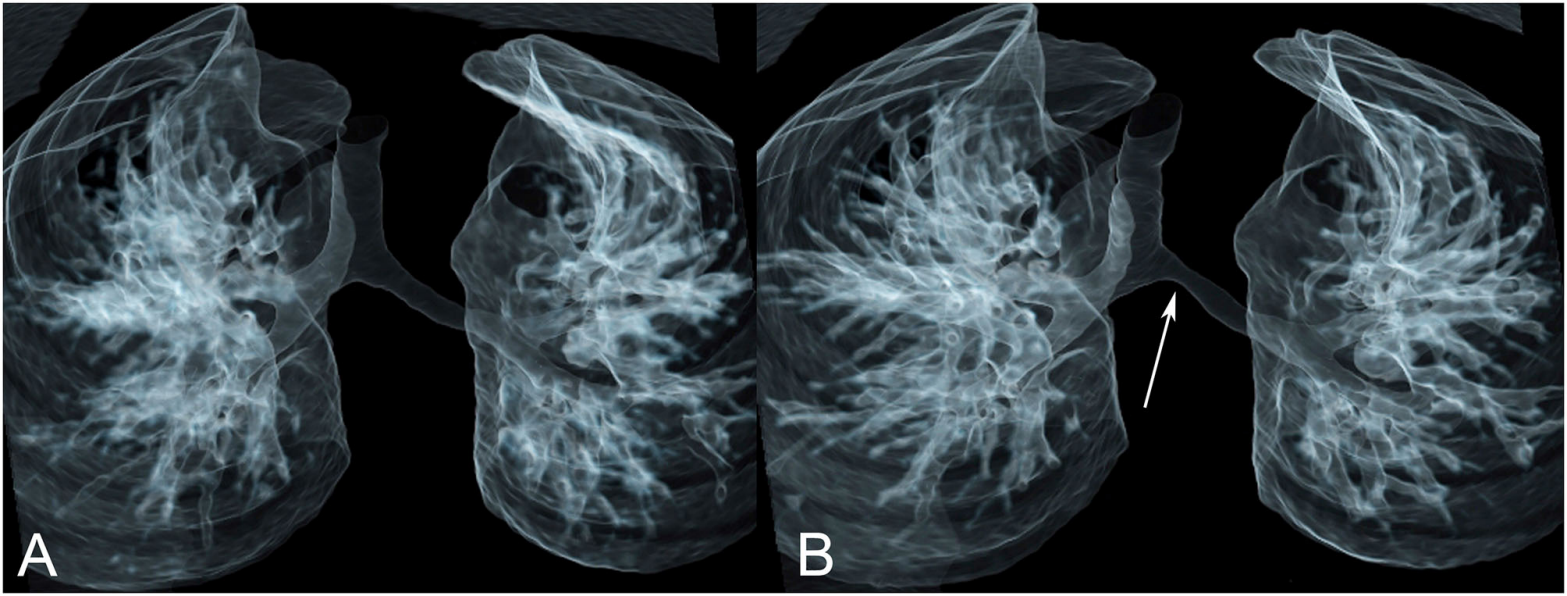
Tracheobronchomalacia. Paired images from a dynamic (“4D”) CT study in inspiration **(A)** and expiration **(B)** show focal collapse of the left main bronchus in expiration (arrow).

The diagnosis of tracheobronchomalacia is difficult, and the evidence base for recommendations is quite poor (31). Both bronchoscopy and bronchography involve instrumentation of the airway, and are consequently non-physiological. Advances in CT technology now permit non-invasive imaging of the major airways, with adequate temporal resolution at an acceptable radiation dose. Images can be displayed as cross-sectional slices or so-called “4D” images (Figure 10).

### Small Airway Obstruction

**Figure 11:**
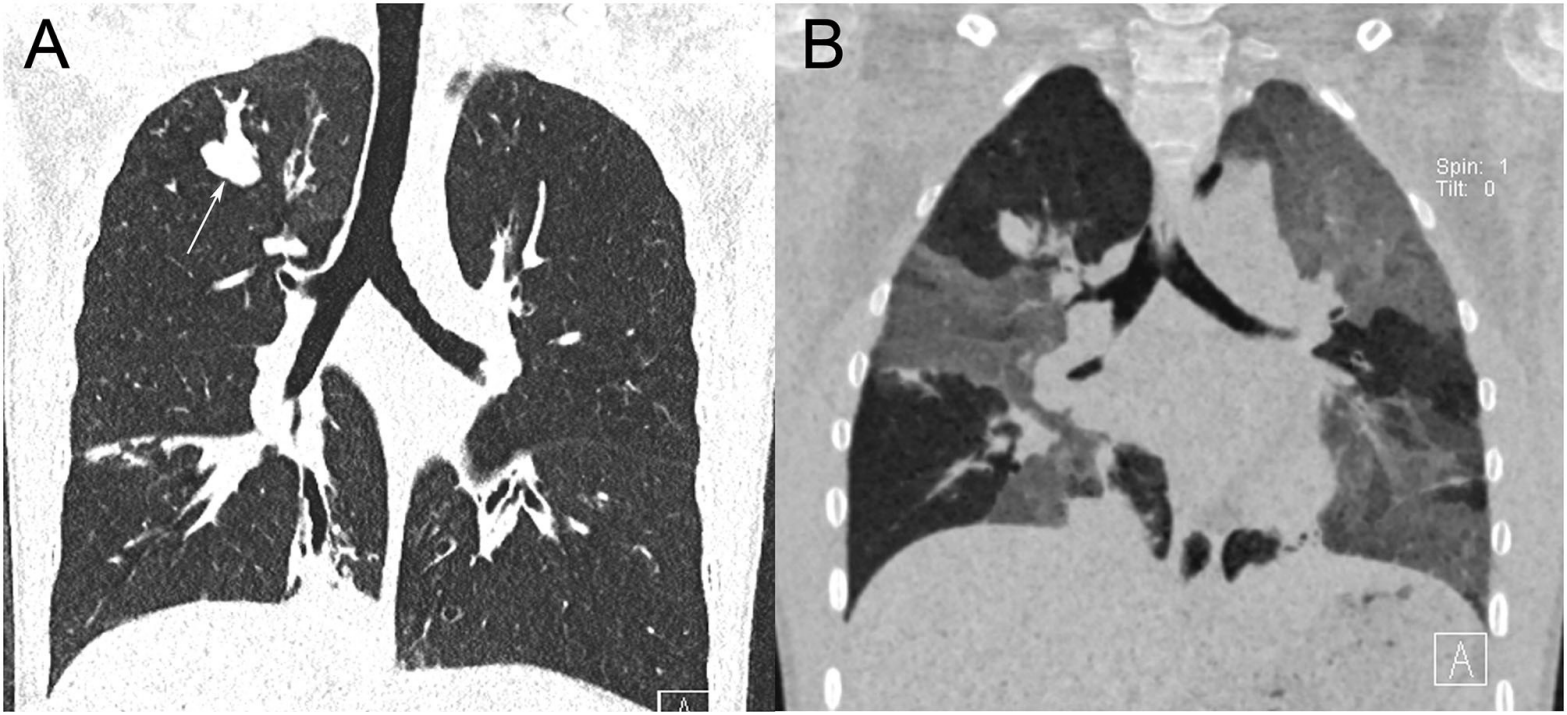
Chest CT in a 13-year-old female with cystic fibrosis. **(A)** Coronal inspiratory CT image shows upper and lower lobe bronchiectasis. A bronchus in the right upper lobe and its branches are markedly distended with a mucus plug (arrow). **(B)** Coronal expiratory minimum intensity projection (MinIP) image shows widespread but patchy air trapping, indicating small airways disease.

The smaller the airway the harder it is to demonstrate airway obstruction directly, and the more we rely on indirect signs, principally air trapping. CT is currently the most useful technique for showing areas of relative hyperinflation of the lung, and is therefore the logical choice when small airway obstruction is suspected (Figure 11).

### Cystic Fibrosis

Traditionally the chest radiograph was the mainstay of imaging in children with cystic fibrosis (CF), but significant reductions in the radiation dose required (and increasing acceptance of low-dose CT images by radiologists) have swung the balance in favor of CT.

CT is the gold standard for the diagnosis and monitoring of bronchiectasis and many studies have validated the utility of CT for CF in particular. In parallel with this, multiple papers have validated the sustained diagnostic performance of CT with ever lower radiation doses on more advanced machines. From these studies it is clear that optimal determination of airway caliber requires volume-controlled (spirometric or equivalent) inspiratory and expiratory scans. Traditional, validated semi-quantitative CT scoring systems are typically employed, though more recently there has been a move toward radiologist-independent quantitative systems to deliver more consistent data (32).

**Figure 12:**
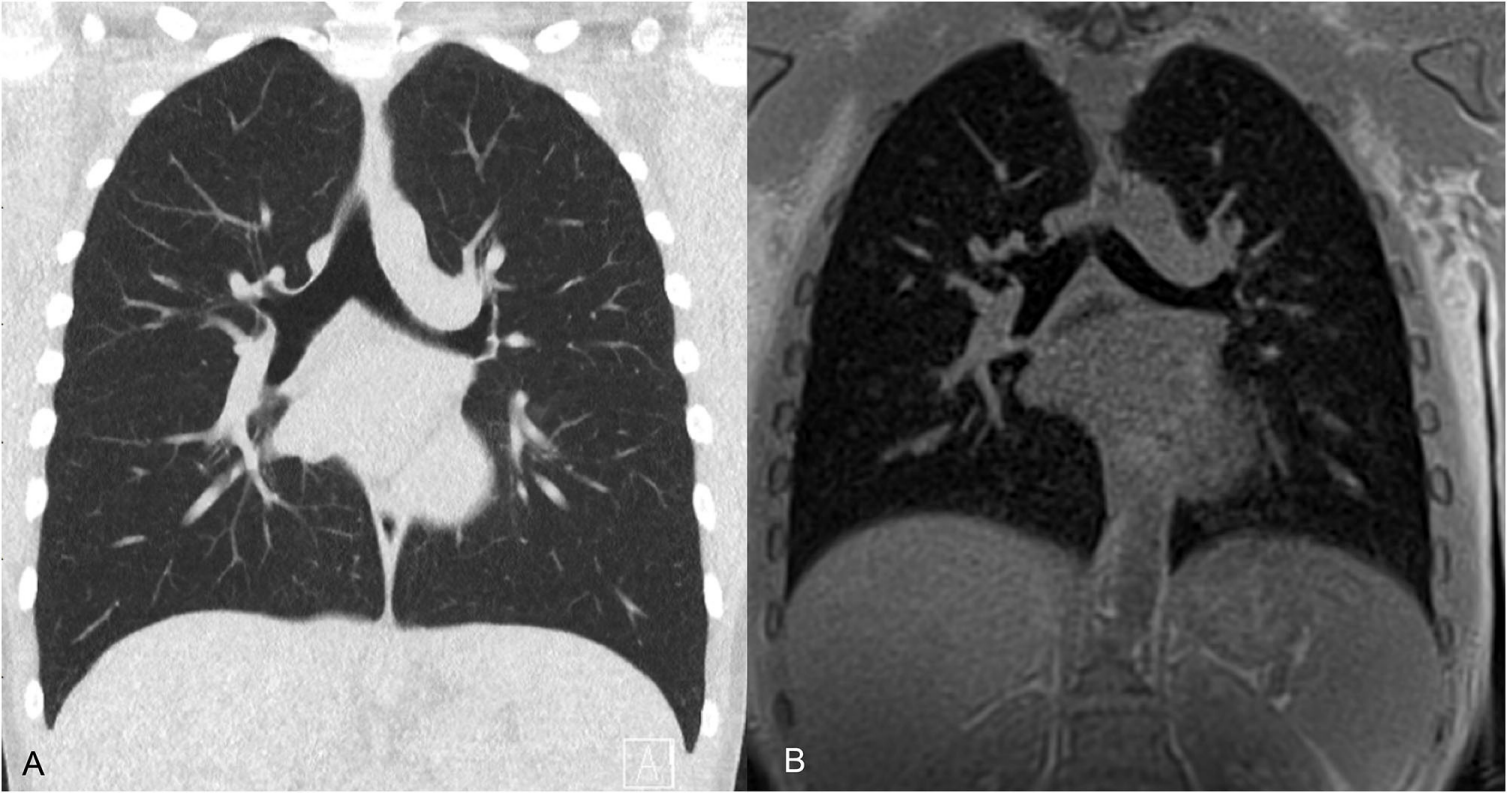
CT and MRI of the airways in a 12-year-old male. **(A)** Coronal 3-mm low dose CT acquired at total lung capacity shows bronchi to the fourth (subsegmental) airway generation. **(B)** MRI averaged over 3 min free breathing, therefore close to functional residual capacity, shows bronchi to the third (segmental) airway generation.

The use of MRI to show direct and indirect features of airways imaging is nascent, not least because of the image-destroying nature of air in motion. Newer ultra-fast MRI techniques partially avoid this issue such that smaller airways can be visualized (Figure 12), yielding a CT-like appearance. Further research is needed to establish the utility of MRI for the monitoring of the anatomic changes of CF.

Newer MRI techniques can demonstrate the decreased overall ventilation and upper lobe predominant ventilation defects in patients with CF (33), and an MRI scoring system has been shown to correlate with FEV_1_ (34). It has been suggested that certain MRI findings may be useful predictors of deterioration in FEV_1_ in children with CF (34), but whether these add clinically useful information to the known non-imaging predictors (35) is an open question.

### Asthma

Chest radiographs and CT may both have a role in the management of selected children with acute asthma, because they may detect complications such as air leaks or associated conditions such as allergic bronchopulmonary aspergillosis or eosinophilic granulomatosis with polyangiitis. Despite significant advances in both CT and MRI techniques, currently neither has a role in the routine management of the chronic disease (36). The appearance of asthma on CT, including bronchial wall thickening, bronchiectasis, endobronchial secretions, air trapping and atelectasis, is indistinguishable from bronchitis (37). These findings have, however, not been consistently reported across the literature.

## Conclusion

Various imaging techniques may be used to diagnose airway obstruction in children. The choice of technique will depend on the level and nature of suspected obstruction, as well as patient-specific factors such as age and ability to cooperate.

## Author Contributions

DR wrote the draft manuscript, which was reviewed and revised by CM and CAM. DR and CAM prepared the images. All authors contributed to the article and approved the submitted version.

## Conflict of Interest

The authors declare that the research was conducted in the absence of any commercial or financial relationships that could be construed as a potential conflict of interest.
